# Fabrication of Nose to Brain Delivery of Pterostilbene‐Loaded Thermoresponsive Mucoadhesive Nanoemulgel to Mitigate Vascular Dementia

**DOI:** 10.1002/alz70859_101945

**Published:** 2025-12-25

**Authors:** Vishal Shivaji Patil, Bhaskar Jyoti Dutta, Sanjiv Singh

**Affiliations:** ^1^ National Institute of Pharmaceutical Education and Research, Hajipur, Bihar India

## Abstract

**Background:**

Vascular dementia (VaD), a heterogeneous neurocognitive disorder, arises from cerebrovascular pathology that impairs cerebral blood flow, causing progressive cognitive decline. It is the second most common form of dementia globally, after Alzheimer’s disease. Pterostilbene (PTE), a natural stilbene derivative, has shown promise as a therapeutic candidate for VaD due to its antioxidative, anti‐inflammatory, and neurogenic properties. However, its clinical utility is limited by poor water solubility and low bioavailability.

**Method:**

To address these limitations, a PTE‐loaded nanoemulsion (PNE) was developed and converted into a thermoresponsive mucoadhesive nanoemulgel (PNEG) for nasal delivery to facilitate direct brain transport. PNE was formulated using the aqueous microtitration method with medium‐chain triglycerides as the oil phase, Tween 80 as the surfactant, and PEG 200 as the co‐surfactant. Chitosan and poloxamer 407 were incorporated into PNEG as mucoadhesive and thermoresponsive polymers, respectively. The formulations were characterized for particle size, zeta potential, encapsulation efficiency, morphology, mucoadhesive strength, in vitro drug release, and ex vivo nasal permeation. Cellular toxicity and uptake studies were conducted in RPMI 2650 and SH‐SY5Y cell lines, and efficacy was evaluated in a rat model of vascular dementia induced by bilateral common carotid artery occlusion (BCCAO).

**Result:**

PNEG demonstrated favorable physicochemical properties, including a particle size of 76.49 ± 0.35 nm, zeta potential of 14.97 ± 3.58 mV, PDI of 0.2704 ± 0.0123, and strong mucoadhesive strength (5.63 g). Transmission electron microscopy revealed a uniform spherical nanoparticle morphology. Cytotoxicity and cellular uptake studies confirmed the safety and efficacy of the formulations. In vivo studies showed significant cognitive improvement in PNE‐ and PNEG‐treated VaD rats, as assessed by the Morris Water Maze and Novel Object Recognition Test. Both formulations reduced oxidative stress, hippocampal apoptosis, and neuroinflammation, with PNEG exhibiting superior efficacy via modulation of the SIRT1/Nrf2/HO‐1 pathway.

**Conclusion:**

PNEG is a promising therapeutic strategy for vascular dementia, addressing the limitations of conventional formulations and enhancing brain bioavailability. This approach offers potential for improved management of VaD through targeted neuroprotection and cognitive preservation.

